# Characterization of *Zygosaccharomyces lentus* Yeast in Hungarian Botrytized Wines

**DOI:** 10.3390/microorganisms11040852

**Published:** 2023-03-27

**Authors:** Hajnalka Csoma, Lajos Acs-Szabo, László Attila Papp, Zoltán Kállai, Ida Miklós, Matthias Sipiczki

**Affiliations:** 1 Department of Genetics and Applied Microbiology, University of Debrecen, 4032 Debrecen, Hungary; 2 Research Institute for Viticulture and Oenology, Tarcal, Department of Oenological Microbiology, University of Debrecen, 4032 Debrecen, Hungary

**Keywords:** *Zygosaccharomyces*, spoilage yeast, botrytized wine, post-fermentation

## Abstract

Tokaj botrytized sweet wines are traditionally aged for several years in wood barrels or bottles. As they have significant residual sugar content, they are exposed to microbial contamination during ageing. Osmotolerant wine-spoilage yeasts are most commonly found in the Tokaj wine-growing region in the species *Starmerella* spp. and *Zygosaccharomyces* spp. For the first time, *Z. lentus* yeasts were isolated from post-fermented botrytized wines. Our physiological studies confirmed that these yeast strains are osmotolerant, with high sulphur tolerance and 8% *v*/*v* alcohol tolerance, and that they grow well at cellar temperature in acidic conditions. Low β-glucosidase and sulphite reductase activities were observed, whereas protease, cellulase, and α-arabinofuranosidase extracellular enzyme activities were not detected. Molecular biology analyses carried out by RFLP analysis of mtDNA revealed no remarkable differences between strains, while microsatellite-primed-PCR fingerprinting of the (GTG)_5_ microsatellite and examination of chromosomal pattern revealed considerable diversity. The fermentative vigour of the tested *Z. lentus* strains was found to be significantly lower compared to the control *Saccharomyces cerevisiae* (Lalvin EC1118). It can be concluded that *Z. lentus* is a potential spoilage yeast in oenology which may be responsible for the initiation of secondary fermentation of wines during ageing.

## 1. Introduction

In certain areas of the world, particular conditions permit *Botrytis cinerea* to cause the noble rot of mature grapes. Due to the specific local climatic conditions characterized by an alternation between short humid periods (favouring the conidial germination and invasive growth of *Botrytis*) and longer dry periods, the *Botrytis* fungi penetrate the grape skins with mycelia to feed and take water from the grapes. The mature grapes lose much of their water content and their chemical composition changes too. This process is called noble rot. The resulting shrivelled berries are used for producing some of the most famous dessert wines of the world, like Tokaj Aszú and Sauternes. The best-known regions that produce the greatest botrytized white wines are Sauternes-Barsac (France), Rheingau and Mosel-Saar-Ruwer (Germany), and Tokaj (Hungary), with the longest documented tradition in Tokaj [1,2].

By far the most specific Tokaj wine is “Essence” or “Eszencia”, possibly one of the most precious wines in the world. The Essence is made from noble-rotted berries, which are harvested selectively from the *Botrytis*-infected bunches and stored in huge vats for a couple of days. During storage, a small amount of juice seeps from the berries under the grapes’ own weight. This juice is called “Essence”. The collected Essence is stored in glass balloons for fermentation and ageing (usually at temperatures below 13 °C). Because of the low temperature and the very high sugar content (in the best years, it may reach 850 g/L), fermentation proceeds very slowly, and the final alcohol level is usually less than 8% *v*/*v*.

Another Tokaj wine specialty called Tokaj Aszú should possess a minimum sugar and extract contents (120 g/L sugar and 35 g/L sugar-free extracts); besides that, the minimum alcohol content was determined to be 9% *v*/*v* (Hungarian Regulation, 2013). Like other botrytized wines, it has remarkable ageing potential. Most improve with several months to years of in-barrel maturation, followed by many years of in-bottle ageing. In Tokaj, at least an 18-month barrel-ageing period is compulsory for Aszú wines. Other botrytized wines are produced similarly, but they are not barrel-matured and can be labelled as “late harvest”, but not Tokaj Aszú [3]. However, storage is always in cold cellars below 13 °C.

Species of the genus *Saccharomyces*, *Candida*, *Kluyveromyces*, *Hanseniaspora*, *Pichia*, and *Zygosaccharomyces* may contribute significantly to the fermentation of botrytized wines [1,2,4,5,6]. The yeast species evinced previously from Tokaj Essences were *Zygosaccharomyces bailii*, *Z. rouxii*, *Z. lentus*, *Z. bisporus*, *Z. pseudobailii*, *Starmerella bacillaris*, *S. lactis-condensi*, *S. cerevisiae*, *Metschnikowia pulcherrima*, *P. membranifaciens, C. apicola, K. fluxuum*, and *H. osmophila* [7,8]. *S. cerevisiae*, *S. uvarum*, *S. bacillaris*, and *Zygosaccharomyces* spp. strains were detected in the case of Tokaj Aszú wines [9,10,11,12].

*Zygosaccharomyces* species are also present naturally during grape juice fermentation [13,14] and some strains possess characteristics useful for winemaking [15,16,17,18]. However, the presence of *Zygosaccharomyces* constitutes an indicator of future spoilage problems, despite the eventual capacity of conferring to the wine useful organoleptic properties [19]. Botrytized sweet wines can take up to years to mature, traditionally in wooden barrels or bottles. In the post-fermentation period, the yeast *Z. bailii* may retain its activity [20] and there is a risk of re-fermentation [21,22].

This genus includes some of the most osmotolerant organisms known, yeasts that can resist concentrations of food preservatives vastly more than those normally encountered [23,24]. Thus, products with low pH or high sugar concentration, such as soft drinks and fruit juices or wine, are at risk of deterioration caused by *Zygosaccharomyces*.

Spoilage results in yeast clouds, particulates, off-flavours, odours, and excessive gas production. In the food and drink industries, the most problematic spoilage yeasts encountered are those belonging to the genus *Zygosaccharomyces* [25]. The genus *Zygosaccharomyces* includes *Z*. *bailii*, *Z*. *bisporus*, *Z*. *gambellarensis*, *Z*. *kombuchaensis*, *Z*. *lentus*, *Z*. *machadoi*, *Z*. *mellis*, *Z*. *parabaillii*, *Z*. *pseudobailii*, *Z*. *pseudorouxii*, *Z*. *rouxii*, *Z*. *sapae*, *Z. favi*, *Z. seidelii*, and *Z*. *siamensi* [26].

In our previous study, we reported the occurrence of *Z. lentus* strains in a Tokaj Essence, which was in the initial stage of fermentation and not yet considered as wine [8]. For this paper, we isolated, identified, and oenologically characterized *Z. lentus* strains from Hungarian sweet dessert wines. This is the first case where several *Z. lentus* yeast strains isolated from sweet wines in post-fermentation were identified and their molecular and oenological physiological properties were investigated.

## 2. Materials and Methods

### 2.1. Examined Botrytized Wines

One Tokaj Essence (vintage 2002) aged in a glass balloon (50 L) and two Tokaj Aszú (the exact vintage is unknown, before 2013) wines aged traditionally in wood barrels for years in cold cellars were examined in this study. The chemical–analytical parameters of the wines were determined by the (EEC) No 2676/90 standard [27].

### 2.2. Yeasts Isolation from Aszú Wines

After the concentration of 20 mL wine sample by centrifugation (6000× *g*, 10 min), 1 mL samples were taken and streaked onto YPGA (1% yeast extract, 1% peptone, 2% glucose, and 2% agar; all *w*/*v*) plates in duplicates. Individual colonies were isolated from the plates after incubation at 25 °C for 5 days. An Olympus BX40 Fluorescence microscope was used for cell morphology examination of the isolates.

### 2.3. Examined Yeast Strains

Thirty-six *Z. lentus* strains were examined in this study. Of these, 30 strains were isolated from Tokaj Aszú wines; two strains isolated previously from Essence (11-1343, 11-1344) (deposited by Edina Karanyicz into the yeasts culture collection of the Department of Genetics and Applied Microbiology, University of Debrecen, Hungary) and four strains from the CBS collection were used. In addition, two yeast strains of *S. cerevisiae*, one of *Z. bailii* and one of *Z. rouxii*, were used as controls for the different tests. Cultures were maintained on YPGA and in frozen stocks (glycerol, 30% *w*/*v*) at −80 °C. The yeast strains used in this study and their origin are listed in Table 1.

### 2.4. Molecular Taxonomic Identification

DNA was isolated and purified according to the method described in Querol et al. [28]. The D1/D2 domains of the large subunit rRNA genes were amplified from genomic DNA using the PCR conditions described previously [11]. The D1/D2 domains were amplified with primers NL-1 and NL-4 [29].

PCR products were cleaned with a Gel/PCR DNA Fragments Extraction Kit (Geneaid Biotech Ltd., New Taipei City, Taiwan), according to the manufacturer’s introductions. The purified amplicons were directly sequenced by the Microsynth AG sequence service (Balgach, Switzerland). The BLAST network service of the NCBI database (http://ncbi.nlm.nih.gov/blast (accessed on 19 September 2021)) was used for DNA sequence similarity searches. The sequences of the amplified fragments were also compared to the sequences of the type strains obtained from the CBS database using the BLAST2 Sequences tool of NCBI (http://www.ncbi.nlm.nih.gov/blast/bl2seq/wblast2.cgi/ (accessed on 19 September 2021)).

### 2.5. Molecular Typing of the Strains

Microsatellite-primed PCR (MSP-PCR) fingerprinting was carried out with oligonucleotide primer (GTG)_5_. A set of PCR reactions were carried out in microtubes containing a master mix with a final volume of 25 μL, containing 2 mM MgCl_2_, 1 mM dNTPs (Thermo Scientific, Waltham, MA, USA), 25 pM of primer, 1.5 U DreamTaq^®^ DNA Polymerase (Thermo Scientific) with DreamTaq buffer (Thermo Scientific) and sterilized distilled water up to final volume. For each reaction, 10 ng genomic DNA was used. Touchdown PCR was carried out using iCycler (Biorad, Hercules, CA, USA) thermal cycler [5 min at 94 °C; 10 cycles of 30 s at 94 °C, 30 s at Tm + 10 °C (1 °C decrease per cycle until Tm is reached) and 30 s at 72 °C; 35 cycles of 30 s at 94 °C, 30 s at 55 °C and 30 s at 72 °C; 2 min at 72 °C].

Determination of mtDNA restriction patterns was carried out according to Querol et al. [28]. DNA samples were digested using the restriction endonucleases *Hinf*I, *Hae*III, and *Rsa*I (Thermo Scientific) according to the manufacturer’s instructions. Mitochondrial restriction fragments were separated on 1.4% (*w*/*v*) agarose gels in 1 × TBE buffer and stained with ethidium bromide and visualised under UV light.

Yeast chromosomal DNA was prepared in plugs as previously described in Sipiczki et al. [10]. The plugs were loaded in a 1% (*w*/*v*) agarose gel (Bio-Rad) and electrophoresis was performed using the CHEF-Mapper system (Bio-Rad Laboratories, Hercules, CA, USA) under the following conditions: constant voltage of 6 V/cm with 60–120 s switch time ramp at an angle of 120° for 26 h at constant temperature (14 °C). After staining the gel with ethidium bromide, bands were visualised under UV light and photographed. *S. cerevisiae* (S288c) chromosomes were used as molecular marker.

### 2.6. Physiological Characterization of Z. lentus Strains

#### 2.6.1. Assessing the Assimilation of Different Carbon and Nitrogen Sources

The assimilative abilities of carbon (10 compounds) and nitrogen (potassium nitrate and lysine) sources, also used in the classical identification of yeasts, were investigated as described by Barnett et al. [30] in the case of the strains isolated from Tokaj wines.

#### 2.6.2. Phenotypic Characterization via Drop Tests

For all assays, yeast strains were pre-cultivated on YPG agar medium for 2 days at room temperature. Aliquots (10 μL) of the cell suspensions (10^6^ cells/mL) prepared in sterile water were dropped on YPG Agar medium, with pH adjusted to 3.5, containing increasing doses of potassium bisulphite (700, 800, 900 mg/L), ethanol (6, 8, 10, 12% *v*/*v*), and sugar (50, 60, 70%, of total sugar, in which glucose and fructose are in equal proportions). We tested whether the isolates grow in a pH range of 3.5 in a normal YPGA medium at 10 °C. YPGA plates, without a stress agent (pH 6.8), were used as control. All plates were incubated aerobically and checked for 4 to 7 consecutive days for colony development at 24 °C.

The qualitative examination of the ability of organic acid production was examined on calcium carbonate agar at 24 °C for 7 days [31]. The acid-producing ability was deduced from the size of the dissolution zone.

The control strain was *S. cerevisiae* commercial starter culture (Lalvin EC1118). The experiments were carried out in duplicate for each strain in two independent experiments.

#### 2.6.3. Screening Enzymatic Activities

Qualitative determinations of enzymatic activities such as amylase, cellulase, and glycosidase were assayed as in Ganga and Martínez [32], with modification: all media had pH 3.5–5.0 and yeasts were incubated for 4 and 7 days at 24 °C. Synthesis of β-glucosidase was induced by the presence of cellobiose in the medium as described in Arévalo-Villena et al. [33]. Sulphite reductase activity (H_2_S production ability) was determined on BiGGY indicator agar (OxoidUnipath Ltd., Hampshire, UK) at 24 °C for 4 and 7 days. On this medium, H_2_S-positive strains show brown or black colonies, while H_2_S-negative colonies are white. The H_2_S produced was evaluated based on the colours of the colonies.

Proteolytic activity was evaluated by spotting yeast strains on skim milk agar medium. The appearance of a clear zone around the colony, after 7 days at 24 °C, would associate to protease activity [34].

For all assays, 10^6^ cells/mL of yeast suspension were spotted onto the mediums. The control strain was *S. cerevisiae* (S288c). All measurements were performed in duplicate.

#### 2.6.4. Oenological Characterization

Fermentative vigour was measured in 100 mL of synthetic must (10% glucose, 10% fructose, 0.5% KH_2_PO_4_, 0.04% MgSO_4_·7H_2_O, 0.1% vitamin solution [35], and 0.1% yeast extract, pH 3.5 [adjusted with tartaric acid]; all *w*/*v*). The initial yeast inoculums were 10^6^ cells/mL from 2 days synthetic must cultures. Erlenmeyer flasks provided with bubbling CO_2_ outlet were incubated at 13 °C without shaking. On every 5th day, the weight loss caused by CO_2_ production was determined; the fermentative vigour was expressed as g of CO_2_/100 mL of must. After 43 days, the musts were filtered and oenochemical analyses were performed by (EEC) No 2676/90 standard [27]. Fermentative vigour was determined from the average of three replicates.

SO_2_ tolerance was determined in the same must. After autoclaving, must was supplemented with potassium metabisulphite, and the free SO_2_ content was adjusted by titration (OIV-MA-AS323-04A:R2009 standard) [36] to 50 mg/L at 13 °C. Flasks were inoculated in duplicate with 10^6^ cells/mL of 2-day precultures (precultures grown in synthetic must were centrifuged before inoculation) and incubated at 13 °C without shaking for 14 days; two independent experiments were carried out. The flasks were provided with bubbling CO_2_ outlet and the weight loss caused by CO_2_ production was measured every day. Lalvin EC1118-inoculated musts were used for reference. The same experiment, but using sulphur-free must, served as a control.

The ability of yeast strains to grow in the presence of ethanol was also examined. Ethanol was added to synthetic must after autoclaving to obtain media with 0, 5, 8, 10, and 14% (*v*/*v*) ethanol concentrations (in a volume of 50 mL). The cultures were inoculated to give 10^6^ cells/mL from 2-day synthetic must cultures after centrifugation. The incubation was carried out under static conditions for 3 weeks at a controlled temperature of 13 °C. The increase in cell number was measured by optical density using Beckman DU-50 spectrophotometer at 600 nm. Experiments were carried out in duplicate. The same experiment, but using alcohol-free must, served as a control.

The cell growth/fermentation vigour was determined by calculation of the ratio (%) between the growth/fermentation vigour of the isolates in synthetic must with and without ethanol or free SO_2_ during the incubation times. Isolates, with a percentage of growth ratio < 10%, were considered not resistant [34].

### 2.7. Data Analyses

Gel images of the microsatellite patterns were processed using the GelCompare II (version 6.6.11.; Applied Maths, Sint-Martens-Latem, Belgium). The dendrogram was generated using the Dice’s correlation similarity coefficient and the Unweighted Pair Group Method using the Arithmetic means (UPGMA) clustering method. The data on mtDNA-RFLP (concatenation of the data derived from the *Hinf*I, *Hae*III, and *Rsa*I mtDNA-RFLP patterns) and CHEF patterns were coded as binary tables, with 1 representing the presence of a fragment and 0 representing the absence of a fragment. Distances of the band patterns were calculated with the Dice’s coefficient using the UPGMA algorithm. Dendrograms were created with the FigTree version 1.4.3 software (http://tree.bio.ed.ac.uk/software/figtree (accessed on 10 February 2022)).

Statistical analyses of the growth kinetics and physicochemical parameters of the fermentations were performed using the Past 3.14 software package (https://folk.uio.no/ohammer/past/ (accessed on 23 May 2022)) [37]. The data normality was determined with the Shapiro–Wilk W and the Anderson–Darling A tests. The Tukey test for *p* < 0.05 was used to establish statistical differences by one-way analysis of variance (ANOVA) in the case of normally distributed data.

## 3. Results

### 3.1. Examined Botrytized Wines

Two Aszú wines underwent a sluggish secondary fermentation in the barrels. The alcohol content of TK1-Aszú was relatively high and the sugar content was lower due to the secondary fermentation. Essence, of which two yeast strains have been identified previously as *Z. lentus* and deposited in our Strain Collection, showed activity indicative of microorganisms, had over 10 years of ageing, and had an alcohol content of over 5% *v*/*v*. The free SO_2_ content of the wines was dangerously low (Table 2).

### 3.2. Yeast Strains Isolated from Aszú Wines

In wood barrels, aged TK1-Aszú had 7.5 × 10^2^ CFU/L and TK2-Aszú had 8 × 10^3^ CFU/L. After the incubation, the yeast colonies were morphologically described and investigated microscopically, and all the individual yeast colonies (10 from TK1-Aszú and 20 from TK2-Aszú) were isolated. Both wine samples showed the presence of mould and bacteria.

Every yeast colony (20 strains) isolated from TK2-Aszú had a smooth surface and their cells occur singly or in pairs, like the two examined strains from Essence. In contrast, 80% of the colonies isolated from the TK1-Aszú wine had a rough surface and their cells formed star-shaped clumps. In the rest of the TK1 strains, morphology typical for TK2 was observed.

### 3.3. Molecular Identification

Molecular identification of all yeast strains isolated from Aszú wines (30 strains) and those isolated from Essence (2 strain) (Table 1) was performed. The BLAST similarity search with the sequences obtained identified numerous highly similar sequences deposited in databases such as *Z. lentus* strains. Similarity values to the sequence of *Z. lentus* (CBS 8574^T^, KY110247) type strain were between 99% and 100%. The strains differed by one or zero nucleotide substitutions (in some cases with one indel at the beginning of the sequence) in their D1/D2 LSU rRNA gene. Because of the identity between the sequences examined, only two sequences have been deposited in the NCBI GenBank (the sequence of strain 10-1406, accession number OP888097, and the sequence of strain 10-1412, accession number OP888098).

### 3.4. Molecular Typing of the Strains

**Figure 1:**
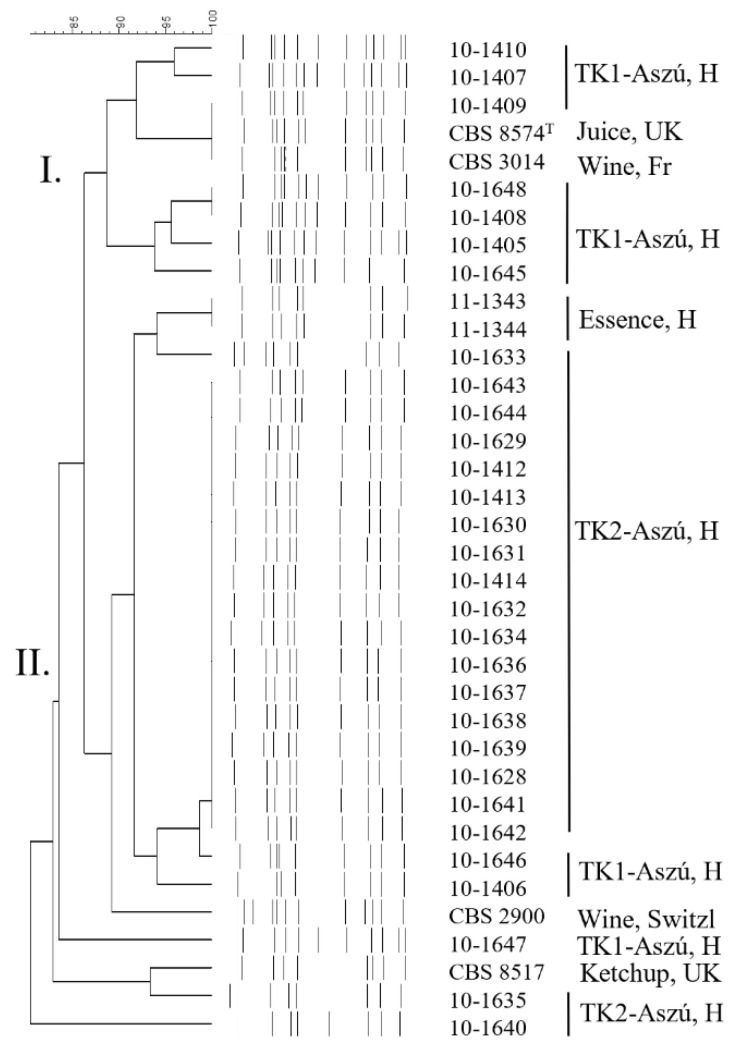
Dendrogram obtained from analysis of the microsatellite profiles using (GTG)_5_ marker constructed with UPGMA. Clusters are indicated by Roman numerals.

Amplification with Microsatellite primer (GTG)_5_ generated complex banding patterns, with 8–12 electrophoretic bands, ranging in size from 250 to 5000 bp. Data showed a total of 16 patterns for the 36 examined *Z. lentus* strains (our isolates and the four collection strains) (Table S1). The strains, except 10-1640, were grouped in two main clusters on the UPGMA dendrogram at a similarity level of 89% (Figure 1). Seventeen strains isolated from TK-2 Aszú wine in cluster II possessed similar band patterns.

Microsatellite PCR revealed a remarkably high genetic diversity, but no correlation with the source or geographical origin of isolation was found.

**Figure 2:**
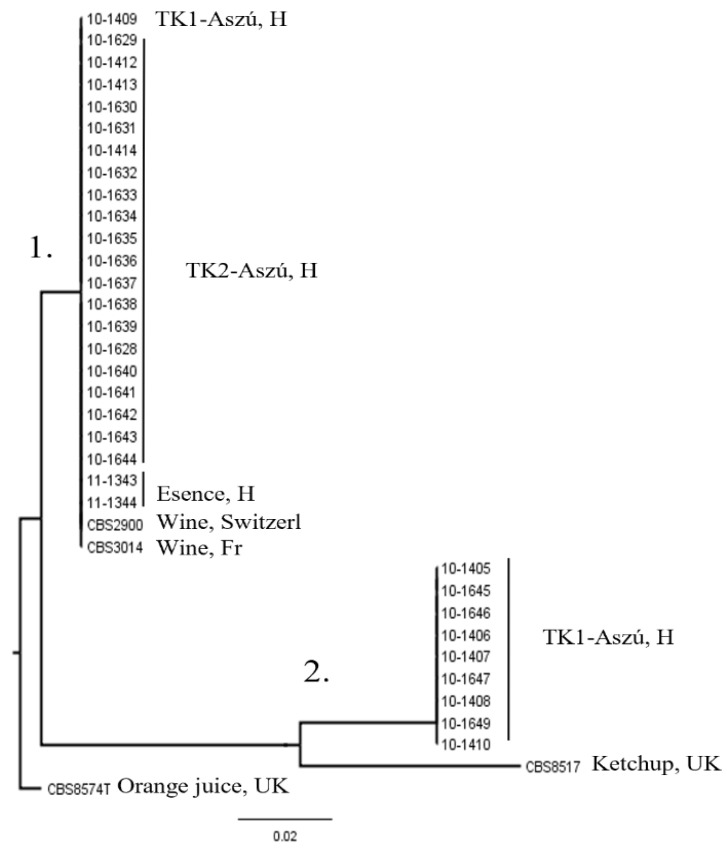
Dendrogram tree built using UPGMA clustering of the concatenated mtDNA-RFLP matrices. Numbers 1 and 2 mark the two clusters.

The examination of the genetic variability of the 36 *Z. lentus* strains by utilizing of mtDNA restriction analysis yielded a low level of polymorphisms. *Hinf*I and *Hae*III were generating four distinct patterns, while *Rsa*I resulted in three (Table S1). The relationship amongst these strains according to the mtDNA-RFLP pattern was evaluated using cluster analysis. The UPGMA dendrogram grouped the strains in two main clusters, and the type strain CBS 8574^T^ is separate from these (Figure 2).

The dendrogram differed from that deduced from the (GTG)_5_-PCR analysis. The isolates from TK-1 Aszú wine (except 10-1409) and CBS 8517 formed a well-separated cluster from the other strains. The mtDNA-RFLP profiles of the distinct groups show a correlation with the source of isolation.

**Figure 3:**
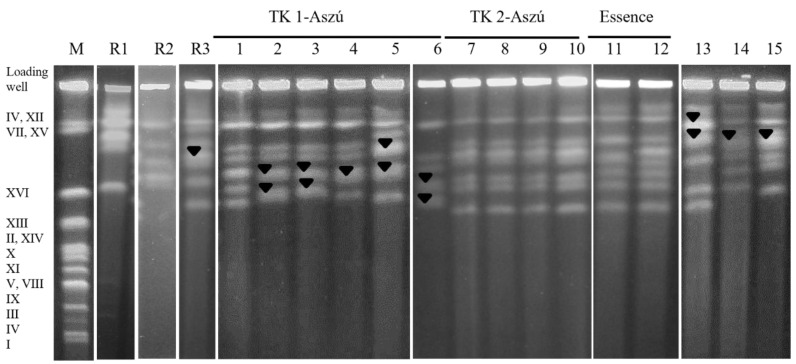
Comparative electrophoretic karyotyping patterns of 11 *Z. lentus* strains analysed. Lane M CHEF marker *S. cerevisiae* S288.c; lane R1 *Z. rouxii* CBS 732^T^; lane R2 *Z. bailii* CBS 680^T^; R3 *Z. lentus* CBS 8574^T^ from orange juice, UK; lanes 1-6 strains 10-1405, 10-1406, 10-1407, 10-1408, 10-1409, and 10-1410 from TK 1-Aszú wine, Tokaj; lanes 7-10 strains 10-1412, 10-1413, 10-1414, and 10-1628 from TK 2-Aszú wine, Tokaj; lanes 11-12 strains 11-1343 and 11-1344 from Essence, Tokaj; lane 13 strain CBS 8517 from ketchup, UK; lane 14 strain CBS 3014 from wine, France; lane 15 strain CBS 2900 from wine, Switzerland. Bands showing double thickness are indicated with a black triangle. Chromosomes are labelled with Roman numerals according to the *S. cerevisiae* nomenclature.

The electrophoretic karyotypes of the 36 *Z. lentus* strain analysed revealed 12 different chromosomal patterns (Table S1). The number of chromosomes may range from six to eight (Figure 3).

The examined strains possess chromosomes approximately between 2200 Kb and 1100 Kb. The bands showing double thickness in CHEF profiling correspond to two chromosomes of the same size (Figure 3). The strains isolated from TK-2 Aszú wine and CBS 2900 have the same karyotype. The electrophoretic karyotyping showed polymorphism amongst the tested strains, which is reflected in the number and size of the chromosomes.

However, in karyotyping, the CBS 8517 strain isolated from ketchup was separated from the others, while in mtDNA analysis, the type material from orange juice was positioned separately.

**Figure 4:**
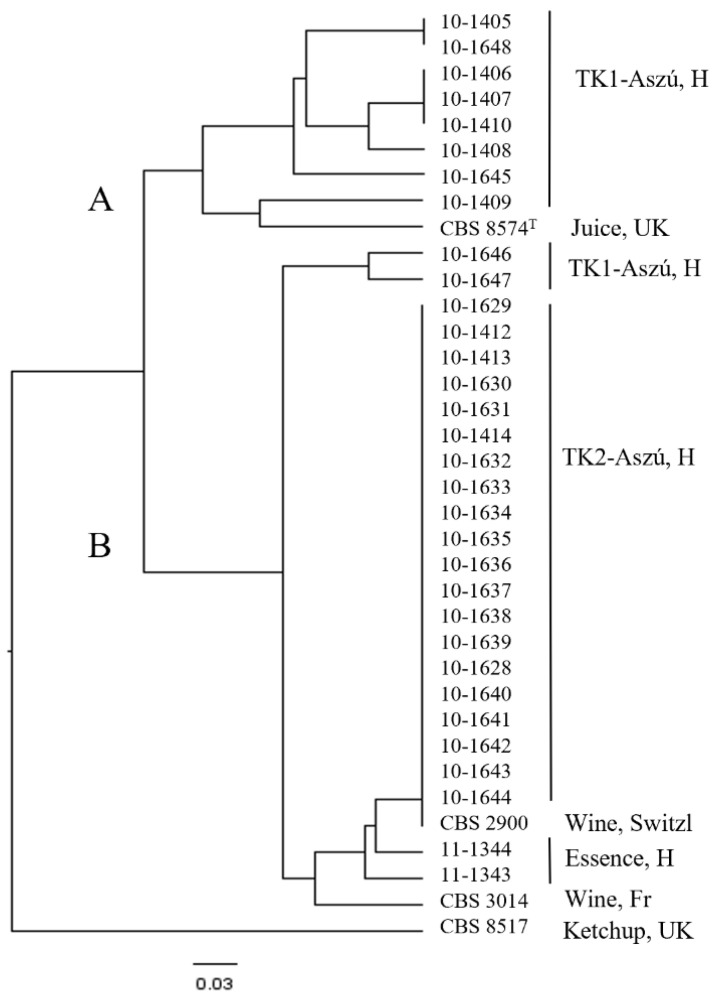
UPGMA dendrogram generated from the CHEF karyotyping of the *Z. lentus* strains. Letters (**A**) and (**B**) mark the two clusters.

The strains isolated from TK2-Aszú wine 10-1412, 10-1413, 10-1414, 10-1628, 10-1629, 10-1630, 10-1631, 10-1632, 10-1634, 10-1636, 10-1637, 10-1638, 10-1639, 10-1641, 10-1642, 10-1643, and 10-1644 have the same molecular profile, which may refer to clonal relationships among these strains. This can also be seen in the UPGMA analysis shown in Figure 4, where the mentioned clonal population, the strains isolated from the Tokaj Essence, the French strain, and the Swiss strain are grouped into cluster ‘B’, like the ‘1’ group separated in the mtDNA analysis.

### 3.5. Physiological and Oenological Characterization

To map the physiological properties of yeasts isolated from Tokaj Aszú wines, we first performed assimilation assays of some carbon and nitrogen components, which are also used in classical identification methods. The 30 isolates assimilated glucose and fructose. None assimilated galactose, cellobiose, maltose, and raffinose. All grew on lysine and weakly on potassium nitrate as nitrogen sources after 7 days and weakly in a vitamin-free medium. The assimilation of sucrose, glycerol, and ethanol proved to be variable properties.

Thirty-two *Z. lentus* strains isolated from Tokaj wines and four from other collections were characterized to evaluate their physiological nature and their potential effects in the wine industry. The results of physiological studies do not show as much variability between the strains isolated from each wine as in the case of microsatellite analysis and karyotyping (Table S2). The acid production profiles of these strains, detected on CaCO_3_ agar, were less uniform. Five strains (isolates from TK2-Aszú and 11-1343) produced medium levels, 10 produced medium-high levels, and CBS 2900 produced high levels. The *Z. lentus* strains tested were tolerant to total sulphur levels up to 900 mg/L. At an alcohol content of 8% *v*/*v*, the ability to reproduce is reduced to a minimum. They are osmotolerant yeasts and were able to form colonies at 50% sugar (25% glucose and 25% fructose), although no growth was observed above this level (Table S2). They were able to grow in an acidic medium (pH 3.5) even at low, i.e., cellar temperatures.

To see if any extracellular enzyme activity could be detected in the *Z. lentus* species, we randomly selected isolates from the two Aszú wines and also tested the other strains. As can be seen from Table S3, β-glycosidase activity was weak and somewhat affected by pH. After one week, some of the strains at pH 5.0 had detectable levels similar to the *S. cerevisiae* control strain (S288c). However, in the more acidic pH range of the wine, this characteristic was not pronounced, except for strain 11-1344. Protease, amylase, cellulase, and α-arabinofuranosidase activity were not detected. In our tests, no isolate had an H_2_S production level (sulphite reductase activity) higher than that of the reference (*S. cerevisiae*, S288c): for two strains (CBS 8574^T^, CBS 2900) no H_2_S production was evincible, nine isolates produced low levels, and five produced medium levels.

Eight representative isolates from Tokaj wines, one from France (CBS 3014) and one from Switzerland (CBS 2900), were then chosen to ferment 100 mL synthetic must and determined for several oenological parameters (Table 3), in comparison with the type strain of *Z. lentus* (CBS 8574^T^) and a commercial *S. cerevisiae* strain (Lalvin EC1118). The selection of the strains isolated from the two Aszú wines was based on the results of the physiological studies (Tables S2 and S3).

The fermentation was implemented under the static conditions at low temperature to simulate natural conditions. After 10 days, the fermentative vigour of all the *Z. lentus* isolates increased; still, less than half of the strains had a fermentative vigour at best equal to 50% of that of the commercial *S. cerevisiae* strain. At 43 days, almost all the *Z. lentus* strains had fermentative vigour values between 3 and 6 g of CO_2_/100 mL, significantly lower than that of the *S. cerevisiae* (8.81 g CO_2_/100 mL): 10-1408 had the highest fermentative vigour (6.45 g CO_2_/100 mL) and CBS 8574^T^ had the lowest (3.24 g CO_2_/100 mL). The strength of fermentation was slight and protracted at 13 °C; when we stopped the fermentation experiments, the process was not completed. The reducing sugar content of the *Z. lentus* samples was between 38–114 g/L. Sugar consumption was significantly lower in all the *Z. lentus* strains except 10-1408 compared to the *S. cerevisiae* strain. The strains originating from other European countries produced a relatively low amount of ethanol (around 5% *v*/*v*), while the majority of the *Zygosaccharomyces* strains isolated from Tokaj wines were able to produce more than 6% *v*/*v*, two strains even 8% *v*/*v*. In addition, strain 10-1408 was the only strain that did not show significant differences compared to the *S. cerevisiae* control in terms of the physicochemical parameters measured. The strains showed relatively high diversity and significant differences in their acetic acid production, which ranged from 0.3 to 1.3 g/L depending on the strain. TK2 strains produce significantly less acetic acid compared to the other strains. The *S. cerevisiae* control strain, except for strains TK2 and 11-1343, did not show a significant difference in acetic acid production compared to the other *Z. lentus* strains. The total titratable acid content of the medium after fermentation was found to be lower in the case of strains isolated from TK2-Aszú wine, like the quantity of acetic acid produced by them. The test on CaCO_3_ agar also proved this. For the other strains, nearly the same total acidity values were measurable as the commercial strain. The pH of the synthetic musts measured after fermentation decreased from 3.5 to 3.2 on average; there was no significant difference between the individual strains.

**Figure 5:**
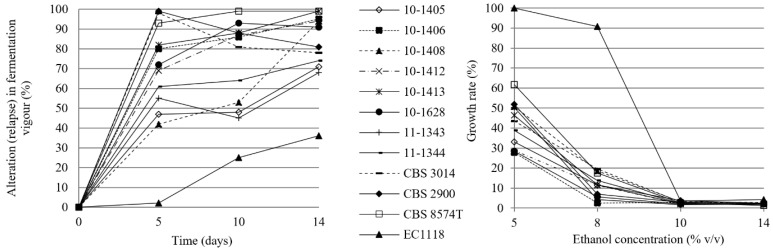
Alteration of the fermentative vigour of the *Z. lentus* strains in the presence of 50 mg/L of free SO_2_ (**left panel**) and growth rate in response to different ethanol concentrations (**right panel**).

The SO_2_ tolerance of the strains was examined in the same kind of must at 13 °C as the fermentative power. All but three strains (CBS 8574^T^, CBS 3014 and CBS 2900) showed a decrease in fermentation vigour compared to sulphur-free fermentation during the first 5 days. (Figure 5). *Z. lentus* isolates 10-1405 and 10-1408 showed a fermentability below 50% in the presence of sulphur but showed an increase by the end of day 14. The strongest inhibition of 50 mg/L free SO_2_ was observed in the case of the commercial culture (Lalvin EC1118). The CBS 8574^T^ was the one with the best SO_2_ tolerance value. On day 10, the titratable free SO_2_ content of the media was 10 mg/l, which is significantly lower than the initial value.

The ability of *Z. lentus* strains to grow at different concentrations of ethanol (5%, 8%, 10%, and 14%, *v*/*v*) during 21 days of incubation at 13 °C was also investigated. The growth was determined by comparing the growth with and without ethanol. Considering ethanol resistance at 13 °C, all the strains grew only at 5% *v*/*v* ethanol (Figure 5). The growth rate of some *Z. lentus* isolates at 8% *v*/*v* of ethanol still reached 10% of that of the control (experiment in alcohol-free synthetic must).

## 4. Discussion

In this work, we report for the first time on the characterization of *Zygosaccharomyces lentus* strains in Hungarian wines. The molecular monitoring of three Tokaj dessert wines by sequencing the D1/D2 domain of the 26S rRNA genes allowed us to detect *Z. lentus* yeast species. Furthermore, we have presented an oenological characterization of 11 *Z. lentus* strains.

Only a few microorganisms can tolerate the harsh, inappropriate environment offered by wine, such as high ethanol concentrations, acidic pH, and the presence of SO_2_, added to low temperatures. The lack of usable carbon sources, oxygen, and nutrients, or the high sugar content, can limit infections and growth. Certain microorganisms can tolerate these conditions, including *Zygosaccharomyces* [38]. Unique tolerances to high sugar content, organic acids, ethanol, and several preservatives characterize the capability of *Zygosaccharomyces* spp. to contaminate and spoil the wine [39]. *Zygosaccharomyces* have been isolated in vineyard and winery equipment, but it is mostly found in grape juice concentrate, or sweetened or dessert wines [2,8,38]. In the vineyard, *Zygosaccharomyces* species have been isolated from overripe, sour rot, and noble rot (“aszú”) damaged grapes [38,40,41,42,43]. In the winery, *Z. bailii* is the most frequently isolated species, followed by *Z. rouxii*, and *Z. bisporous* [44]. Loureiro and Malfeito-Ferreira [38] suggested that detection rates from wine may be less than actual incidences, potentially because of inadequate methods of detection or a slow proliferation until environmental conditions eliminate competitive organisms. Control and early detection are challenging, as one cell in a bottle may eventually cause a spoilage event [45].

The type strain of *Z. lentus* isolated from orange juice, was previously identified as *Z. bailii*. Ever since, it has been isolated in orange squash, tomato ketchup, whole-orange juice, wine, and traditional balsamic vinegar [46]. *Z. lentus* is related closely to *Z. bailii* and *Z. bisporus*, sharing many of their spoilage characteristics, namely osmotolerance and resistance to benzoic and sorbic acids [46]. In contrast to these species, *Z. lentus* displays slow growth under aerobic conditions and is not able to grow at 30 °C. However, it grows well at 4 °C, counter to other spoilage *Zygosaccharomyces* yeasts, which leads to increases in the probability of their presence in chilled products [47]. To date, there is insufficient scientific evidence that *Z. lentus* is a potentially wine-spoilage yeast [48]. In our previous study [8], we reported strains isolatable from Tokaj Essence (3 years old, 1.95% *v*/*v* alcohol content) and now we have detected them in Tokaj Aszú wines undergoing post-fermentation.

In this study, we investigated the morphological, molecular, and physiological diversity of three populations of *Z. lentus* in wine. Morphologically, they could be divided into only two groups. However, molecular tests revealed a significant diversity. The three molecular methods used, RFLPs of mtDNA, MSP-PCR analysis, and electrophoretic karyotyping, have all been applied previously to the comparative study of *Zygosaccharomyces*. The strain-level resolution power of these techniques was proved for the *Zygosaccharomyces* species formerly [19,49,50]. Esteve-Zarzoso et al. [19] compared the mtDNA-RFLP patterns (*Hinf*I) and karyotypes of *Z. lentus* strains (CBS 3014 and CBS 2900) to those of *Z. bailii* strains. The genetic diversity of the *Z. lentus* species remained so far unexplored. The RFLP analysis of the mtDNA proved to be suitable for the fingerprinting of the strains of the *Zygosaccharomyces* genus, even when they were isolated from the same source [19,50]. Our results revealed a high level of similarity in the mitochondrial genome and isolates from wines grouped together irrespective of their geographic origins. Contrary to this, greater heterogeneity was observed in the nuclear genome. Chromosomal karyotyping using different electrophoretic systems are chosen methods to differentiate yeast strains [19,51]. They were proved to be effective in discriminating between *Saccharomyces* strains genetically closely related [52,53] and in typing strains of similar geographical origin [54]. In the case of the genus *Zygosaccharomyces*, species-specific chromosomal patterns were observed [19]. That study has shown that electrophoretic karyotyping tends to reveal differences between species of this genus rather than between the individual strains of the same species. We examined 36 strains to get more information about their chromosome profiles and the efficiency of this method. We conclude that it is not possible to determine a species-pattern characteristic for all strains of the *Z. lentus* species, because of the diversity in the karyotypes of the strains. At the same time, clear strain-patterns were observed within the isolates originating from TK-Aszú wines. One clonal population was evidenced by TK 2-Aszú wine.

In the following, we have tried to find correlations between the molecular diversity of strains and their physiological characteristics, with particular emphasis on those of oenological relevance. For the physiological characterization, parameters of oenological interest were examined and *Z. lentus* isolates were tested qualitatively for total acid and H_2_S production, the presence of enzymatic activities, the growth at different concentrations of ethanol, and SO_2_ and fermentation vigour. The enzymes produced by yeasts during the winemaking process could directly influence the fermentation medium; their effect may be injurious or favourable, depending on the nature of the enzyme and the environmental conditions [32]. The major extracellular enzymes in winemaking include esterase, glycosidase, lipase, β-glucosidase, protease, and cellulase usually involved in the hydrolysis of structural components [55,56,57]. Our studies showed some activity for the extracellular enzymes β-glycosidase and sulphite reductase. The sulphite reductase activity of the Tokaj isolates was moderate to varying degrees compared to *S. cerevisiae* control strain, and in some cases undetectable. The lower H_2_S production capacity can be considered a positive feature. The β-glycosidase activity is pH dependent, only weakly expressed in the lower pH range of wine for the tested strains.

Sulphite (SO_2_) has been extensively used in food and wine processing as an antioxidant and antimicrobial agent [58]. In Hungary, the legal limit of total SO_2_ is at most 400 mg/L and the free SO_2_ is at most 60 mg/L in Tokaj botrytized wine specialities. Tokaj Aszú generally contains a free SO_2_ level lower than German and French versions (20–30 mg/L) [3]. The presence of at least 50 mg/L free sulphites is necessary to terminate fermentation [59,60]. To provide this level, a large amount of SO_2_ must be used, since most of the added sulphites combine with the carbonyl compounds. The chemical and antimicrobial effect of SO_2_ is related to the availability of the molecule and the pH of the wine. In solution (like wine), the free forms of SO_2_ have an antimicrobial effect. In our experiment, it was shown that 50 mg/L free SO_2_ does not effectively inhibit the growth of *Z. lentus* strains.

The screening for resistance at increasing concentrations of ethanol is also important from an oenological point of view. Not every tested strain showed growth in a medium containing 8% *v*/*v* ethanol, which confirms previous observations by Steels et al. [47] that *Z. lentus* tolerates lower alcohol. However, we should note that this property depends on the tested strain. It should also be mentioned that the two Aszú wines serving as the source of isolation contained 8 and 13.4% *v*/*v* ethanol. It follows that the higher alcohol content inhibits the proliferation of the cells but does not kill all of them.

The fermentation capacities of the *Z. lentus* strains were significantly lower than that of the *S. cerevisiae* starter strain (Lalvin EC1118), except for one strain. However, for quite a few of the *Z. lentus* strains, we measured not significantly different amounts of total acidity and acetic acid content as the starter strain (Lalvin EC1118) at the end of the fermentation experiment. An important parameter of wine is the volatile acidity, which usually ranges between 500 and 1000 mg/L, 90% of which is acetic acid (0.2–2.0 g/L) [61]. The permitted limit according to European regulations is 1.2 g/L acetic acid [62], but a concentration above 0.8 g/L acetic acid can impart a harmful vinegar taste [63] to wine. *S. cerevisiae* strains produce acetic acid in the range of 0.3–1.2 g/L [64], but production is nevertheless affected by pH, sugar, and nitrogen concentrations [65]. Domizio et al. [66] found that the *Zygossaccharomyces* genus strains they studied produced a relatively variable amount of acetic acid during fermentation, but this was below the amount measured for *S. cerevisiae* strains (0.9–1.0 g/L). We can draw a similar conclusion for the *Z. lentus* strains we studied.

We did not find a correlation between the genetic and physiological traits of the strains. Strains of similar genetic characteristics were not necessarily physiologically similar to each other. However, there are significant differences in the fermentation and acetic acid production capabilities between TK1 and TK2 isolates.

The results we have obtained reveal that the *Z. lentus* strains are both heterogeneous in terms of their morphological, physiological, and molecular properties. We can consider it a potential polluting yeast, which can survive in the dessert wines we have studied, despite the high ethanol content and the low temperature, and may adversely affect its properties or composition during any secondary fermentation.

Even after barrel ageing and bottling, the wines can be contaminated by lactic acid bacteria, acetic acid bacteria, and yeasts. These micro-organisms metabolise the residual sugars in the wine to produce carbon dioxide, acids from alcohol and metabolites that affect the aroma. It is therefore important to detect infective micro-organisms in time and to develop appropriate defenses against them. Further fermentation experiments are needed to assess the impact of *Z. lentus* yeast strains on the aroma composition of wines.

## 5. Conclusions

1. *Zygosaccharomyces lentus* is a potential contaminant yeast that may persist in the dessert wines we studied, despite high ethanol content and low temperatures, and may cause secondary fermentation during ageing.
2. The results we have obtained reveal that the *Z. lentus* species is heterogeneous in morphological, physiological, and molecular properties.
3. Mitochondrial RFLP appears poorly discriminant at the strain level. Our results revealed a high level of similarity in the mitochondrial genome and isolates from wines grouped irrespective of their geographic origins.
4. In contrast, greater heterogeneity in the nuclear genome was observed in microsatellite analysis and electrophoretic karyotyping.
5. We conclude that it is not possible to determine a species-specific chromosomal pattern characteristic of all strains of the *Z. lentus* species, because of the diversity in the karyotypes of the strains.
6. It was shown that even 50 mg/L of free SO_2_ is not sufficient to inhibit the growth of *Z. lentus*.
7. The fermentation capacities of the *Z. lentus* strains were lower than that of the *S. cerevisiae* starter strain and fermentations were sluggish. Nevertheless, every tested isolate utilized half or more of the starting sugar content and produced varying amounts of alcohol and acetic acid.

## Figures and Tables

**Table 1:** Yeast strains used in this study.

Species	Another Identifier	Source, Country	Yeast Strains ^a^
*S. cerevisiae*	YGSC X4005-11A		S.288c
*S. cerevisiae*	Lalvin EC1118	Wine, France	10-1411
*Z. bailii*	CBS 680^T^	Unknown	10-1428
*Z. rouxii*	CBS 732^T^	Concentrated black grape must	10-514
*Z. lentus*	CBS 8574^T^	Orange juice, UK	10-1430
*Z. lentus*	CBS 2900	Wine, Switzerland	10-1429
*Z. lentus*	CBS 3014	Wine, France	10-1426
*Z. lentus*	CBS 8517	Ketchup, UK	10-1427
*Z. lentus* *		TK1-Aszú wine, Tokaj, Hungary	10-1405, 10-1406, 10-1407, 10-1408, 10-1409, 10-1410, 10-1645, 10-1646, 10-1647, 10-1648
*Z. lentus* *		TK2-Aszú wine, Tokaj, Hungary	10-1412, 10-1413, 10-1414, 10-1628, 10-1629, 10-1630, 10-1631, 10-1632, 10-1633, 10-1634, 10-1635, 10-1636, 10-1637, 10-1638, 10-1639, 10-1640, 10-1641, 10-1642, 10-1643, 10-1644
*Z. lentus*		Essence, Tokaj, Hungary	11-1343, 11-1344

^a^ Culture Collection of the Department of Genetics and Applied Microbiology. University of Debrecen, Hungary CBS. Centraalbureau voor Schimmelcultures. Utrecht, The Netherlands. YGSC. Yeast Genetic Stock Centre. University of California at Berkeley, USA. * Strains isolated in this study.

**Table 2:** Oenological parameters of the examined wines.

Samples	Alcohol	Residual Sugar	Total Acidity	pH	SO_2_		Acetic Acid
					Free	Total	
	(%, *v*/*v*)	(g/L)	(g/L)		(mg/L)	(mg/L)	(g/L)
TK1-Aszú	13.38	114.50	11.80	3.35	6	238	1.12
TK2-Aszú	8.11	283.00	8.20	3.47	5	45	0.93
11-13-Essence	5.26	638.50	9.7	3.32	12	267	1.18

**Table 3:** Fermentative vigour and physicochemical parameters obtained from micro fermentations.

Yeast Strain	Fermentative Vigour ^1^ (5 Days)	Fermentative Vigour ^1^ (10 Days)	Fermentative Vigour ^1^ (43 Days)	Reducing Sugars ^2^ (g/L)	Alcohol ^2^ (%, *v*/*v*)	Total Acidity ^2^ (g/L)	Acetic Acid ^2^ (g/L)
10-1405	0.15 ± 0.02 ^b^	0.97 ± 0.40 ^b^	5.61 ± 0.26 ^b,d,f,h,i,k,n,p,r,s,u^	85.00 ± 8.76 ^b,c,f,h,i,k,m,p,q,s^	8.18 ± 0.34 ^b,d,f,h,j,l,n,p,r,s^	4.46 ± 0.65 ^a^	0.83 ± 0.18 ^a,c,f,g,i,k,n,p,r,s,u^
10-1406	0.15 ± 0.02 ^b^	0.80 ± 0.37 ^b^	5.26 ± 0.15 ^b,d,f,h,i,k,n,p,q,t,u^	106.00 ± 9.0 ^b,c,e,g,i,k,m,o,q,s^	6.18 ± 0.18 ^b,d,e,gi,k,m,o,q,t^	4.24 ± 0.27 ^a^	0.87 ± 0.18 ^a,c,f,g,i,k,n,p,r,s,u^
10-1408	0.21 ± 0.13 ^b^	1.14 ± 0.50 ^b,d^	6.45 ± 0.21 ^b,d,f,h,j,l,n,p,r,s^	38.71 ± 0.92 ^a,d,f,h,j,l,n,p,r^	8.66 ± 0.53 ^a,d,f,h,j,l,n,p,r,s^	4.62 ± 0.33 ^a^	0.99 ± 0.12 ^a,c,e,g,i,l,n,p,r,s^
10-1412	0.20 ± 0.16 ^b^	0.96 ± 0.40 ^b^	4.52 ± 0.13 ^b,d,e,g,i,k,m,o,q^	97.83 ± 8.52 ^b,c,e,g,i,k,m,o,q^	6.40 ± 0.42 ^b,d,e,g,i,k,m,o,q^	3.78 ± 0.39 ^b,c^	0.42 ± 0.12 ^b,d,f,h,j,k,o,q^
10-1413	0.16 ± 0.10 ^b^	0.85 ± 0.20 ^b^	4.19 ± 0.65 ^b,d,e,g,i,l,m,o^	110.33 ± 11.09 ^b,c,e,g,i,k,m,o^	6.10 ± 0.35 ^b,d,e,g,i,k,m,o^	3.86 ± 0.30 ^b,c^	0.33 ± 0.03 ^b,d,f,h,j,k,m,o^
10-1628	0.14 ± 0.10 ^b^	0.72 ± 0.19 ^b^	4.19 ± 0.24 ^b,d,e,g,i,k,m^	101.48 ± 6.35 ^b,c,e,g,i,k,m^	6.25 ± 0.40 ^b,d,e,g,i,k,m^	3.37 ± 0.02 ^b^	0.46 ± 0.08 ^b,d,f,h,j,k,m^
11-1343	0.15 ± 0.09 ^b^	1.07 ± 0.65 ^b^	5.15 ± 0.20 ^b,d,f,g,i,k^	93.58 ± 6.02 ^b,c,e,g,i,k^	6.87 ± 0.21 ^b,d,f,h,i,k^	4.06 ± 0.10 ^a^	0.57 ± 0.11 ^b,c,f,h,i,k^
11-1344	0.17 ± 0.09 ^b^	0.84 ± 0.11 ^b^	4.76 ± 0.73 ^b,d,f,g,i^	95.20 ± 4.30 ^b,c,e,g,i^	6.55 ± 0.27 ^b,d,e,g,i^	4.57 ± 0.04 ^a^	0.86 ± 0.13 ^a,c,f,g,i^
CBS 3014	0.12 ± 0.08 ^b^	0.76 ± 0.30 ^b^	4.25 ± 0.900 ^b,d,e,g^	114.41 ± 13.09 ^b,c,e,g^	5.58 ± 0.22 ^b,c,e,g^	4.26 ± 0.12 ^a,c^	0.92 ± 0.10 ^a,c,e,g^
CBS 2900	0.10 ± 0.00 ^b^	0.55 ± 0.04 ^b,c^	3.69 ± 0.77 ^b,c,e^	109.41 ± 1.28 ^b,c,e^	5.60 ± 0.04 ^b,c,e^	4.70 ± 0.29 ^a^	1.28 ± 0.03 ^a,c,e^
CBS 8574^T^	0.14 ± 0.01 ^b^	0.71 ± 0.17 ^b^	3.24 ± 0.32 ^b,c^	104.25 ± 1.25 ^b,c^	4.77 ± 0.30 ^b,c^	4.86 ± 0.20 ^a^	0.90 ± 0.10 ^a,c^
EC1118	0.88 ± 0.20 ^a^	1.89 ± 0.16 ^a^	8.81 ± 0.62 ^a^	21.26 ± 10.90 ^a,d^	9.35 ± 0.39 ^a^	4.15 ± 0.04 ^a^	1.09 ± 0.08 ^a^

^1^ g CO_2_/100 mL, ^2^ Measured on day 43. All data are expressed as averages (n = 3). Means with the same letter are not significantly different from each other within the same column (Tukey’s test; *p* < 0.05).

## Data Availability

The data presented in this study are available in this manuacript.

## Supplementary Materials

The following supporting information can be downloaded at: https://www.mdpi.com/article/10.3390/microorganisms11040852/s1, Table S1. Summary of the results obtained by the molecular typing methods used. Table S2. Results obtained from the physiological tests of the *Z. lentus* isolates used in this study. Table S3. Extracellular enzyme activity assays of some *Z. lentus* isolates.
