# Supplementary material for: Characterization of Zygosaccharomyces lentus Yeast in Hungarian Botrytized Wines
Source: Microorganisms. 2023 Mar 27;11(4):852. doi: 10.3390/microorganisms11040852 (PMC10145543; doi:10.3390/microorganisms11040852)
Supplement: Supplementary file 1 [file microorganisms-11-00852-s001.zip › Table S1.pdf]

Table S1. Summary of the results obtained by the molecular typing methods used.

Strains	Pattern				
	mtDNA-RFLP			Microsatellite	Karyotype
	*Hinf*I	*Hae*III	*Rsa*I	$(GTG)_5$	
10-1405	A	a	x	1	I
10-1645	A	a	x	2	II
10-1646	A	a	x	3	III
10-1406	A	a	x	4	IV
10-1407	A	a	x	5	IV
10-1647	A	a	x	6	III
10-1408	A	a	x	1	V
10-1409	B	b	y	7	VI
10-1648	A	a	x	1	I
10-1410	A	a	x	8	IV
**10-1629**	B	b	y	9	VII
**10-1412**	B	b	y	9	VII
**10-1413**	B	b	y	9	VII
**10-1630**	B	b	y	9	VII
**10-1631**	B	b	y	9	VII
**10-1414**	B	b	y	9	VII
**10-1632**	B	b	y	9	VII
10-1633	B	b	y	10	VII
**10-1634**	B	b	y	9	VII
10-1635	B	b	y	11	VII
**10-1636**	B	b	y	9	VII
**10-1637**	B	b	y	9	VII
**10-1638**	B	b	y	9	VII
**10-1639**	B	b	y	9	VII
**10-1628**	B	b	y	9	VII
10-1640	B	b	y	12	VII
**10-1641**	B	b	y	9	VII
**10-1642**	B	b	y	9	VII
**10-1643**	B	b	y	9	VII
**10-1644**	B	b	y	9	VII
11-1343	B	b	y	13	VIII
11-1344	B	b	y	13	IX
CBS 8574^T	C	c	y	7	X
CBS 2900	B	b	y	14	VII
CBS 3014	B	b	y	15	XI
CBS 8517	D	d	z	16	XII

The bold-highlighted strains have the same molecular profile.
