# Supplementary material for: Characterization of Zygosaccharomyces lentus Yeast in Hungarian Botrytized Wines
Source: Microorganisms. 2023 Mar 27;11(4):852. doi: 10.3390/microorganisms11040852 (PMC10145543; doi:10.3390/microorganisms11040852)
Supplement: Supplementary file 1 [file microorganisms-11-00852-s001.zip › Table S2.pdf]

Table S2. Results obtained from the physiological tests of the *Z. lentus* isolates used in this study.

Strains	Acid production on $CaCO_3$ agar	Sulphur 700 mg/L	Sulphur 800 mg/L	Sulphur 900 mg/L	Ethanol 6% v/v	Ethanol 8% v/v	Ethanol 10% v/v	Ethanol 12% v/v	Sugar 50%	Sugar 60%	Sugar 70%	10 ºC, YPGA pH 3.5	24 ºC, YPGA pH 3.5	24 ºC, YPGA pH 6.8
10-1405	+	+	+	+	+	w-	-	-	w	-	-	w	+	+
10-1645	+	+	+	+	+	w-	-	-	w	-	-	w	+	+
10-1646	+	+	+	+	+	w-	-	-	w	-	-	w	+	+
10-1406	+	+	+	+	+	w	w-	-	w	-	-	w	+	+
10-1407	+	+	+	+	+	w	-	-	w	-	-	w	+	+
10-1647	+	+	+	+	+	w-	-	-	w	-	-	w	+	+
10-1408	+	+	+	+	+	w-	-	-	w	-	-	w	+	+
10-1409	+	+	+	+	+	w	w-	-	w	-	-	w	+	+
10-1648	+	+	+	+	+	w-	-	-	w	-	-	w	+	+
10-1410	+	+	+	+	+	w-	-	-	w	-	-	w	+	+
10-1629	w	+	+	+	+	w-	-	-	w	-	-	+	+	+
10-1412	w	+	+	+	+	w-	-	-	w	-	-	+	+	+
10-1413	w	+	+	+	+	w-	-	-	w	-	-	+	+	+
10-1630	w	+	+	+	+	w-	-	-	w	-	-	+	+	+
10-1631	w	+	+	+	+	w-	-	-	w	-	-	+	+	+
10-1414	w	+	+	+	+	w-	-	-	w	-	-	+	+	+
10-1632	w	+	+	+	+	w-	-	-	w	-	-	+	+	+
10-1633	w	+	+	+	+	w-	-	-	w	-	-	+	+	+
10-1634	w	+	+	+	+	w-	-	-	w	-	-	+	+	+
10-1635	w	+	+	+	+	w-	-	-	w	-	-	+	+	+
10-1636	w	+	+	+	+	w-	-	-	w	-	-	+	+	+
10-1637	w	+	+	+	+	w-	-	-	w	-	-	+	+	+
10-1638	w	+	+	+	+	w-	-	-	w	-	-	+	+	+
10-1639	w	+	+	+	+	w-	-	-	w	-	-	+	+	+
10-1628	w-	+	+	+	+	w-	-	-	w	-	-	+	+	+
10-1640	w	+	+	+	+	w-	-	-	w	-	-	+	+	+
10-1641	w	+	+	+	+	w-	-	-	w	-	-	+	+	+
10-1642	w	+	+	+	+	w-	-	-	w	-	-	+	+	+
10-1643	w	+	+	+	+	w-	-	-	w	-	-	+	+	+
10-1644	w	+	+	+	+	w-	-	-	w	-	-	+	+	+
11-1343	w	+	+	+	+	w-	-	-	w	-	-	+	+	+
11-1344	+	+	+	+	+	w-	-	-	w	-	-	+	+	+
CBS 8574^T	+	+	+	+	+	-	-	-	w	-	-	+	+	+
CBS 2900	++	+	+	+	+	-	-	-	w	-	-	+	+	+
CBS 3014	+	+	+	+	+	w	-	-	w	-	-	+	+	+
CBS 8517	+	w	-	-	+	w	-	-	w	-	-	+	+	+
EC1118	+	-	-	-	++	++	+	+	w	-	-	+	++	++

Legend	
(white)	-
(light grey)	w-
(grey)	w
(dark grey)	+
(black)	++
