# Supplementary material for: Characterization of Zygosaccharomyces lentus Yeast in Hungarian Botrytized Wines
Source: Microorganisms. 2023 Mar 27;11(4):852. doi: 10.3390/microorganisms11040852 (PMC10145543; doi:10.3390/microorganisms11040852)
Supplement: Supplementary file 1 [file microorganisms-11-00852-s001.zip › Table S3.pdf]

Table S3. Extracellular enzyme activity assays of some *Z. lentus* isolates.

Yeast strains	Extracellular enzymes activity*									
	Protease	Amylase		Cellulase		α-arabinofuranosidase		β-glycosidase		Sulphite reductase
	pH 5.0	pH 3.5	pH 5.0	pH 3.5	pH5.0	pH 3.5	pH 5.0	pH 3.5	pH 5.0	
S288c	+/+	-/+	+/+	-/-	-/-	-/-	-/-	+/+	+/+	5/5
CBS 8574^{T}	-/-	-/-	-/-	-/-	-/-	-/-	-/-	-/w	w/w	1/1
10-1405	-/-	-/-	-/-	-/-	-/-	-/-	-/-	w/w	w/w	3/3
10-1406	-/-	-/-	-/-	-/-	-/-	-/-	-/-	w/w	w/w	2/2
10-1407	-/-	-/-	-/-	-/-	-/-	-/-	-/-	w/w	w/w	2/3
10-1408	-/-	-/-	-/-	-/-	-/-	-/-	-/-	w/w	w/w	2/3
10-1409	-/-	-/-	-/-	-/-	-/-	-/-	-/-	w/w	w/+	3/3
10-1410	-/-	-/-	-/-	-/-	-/-	-/-	-/-	w/w	w/w	3/3
10-1412	-/-	-/-	-/-	-/-	-/-	-/-	-/-	w/w	w/w	4/4
10-1413	-/-	-/-	-/-	-/-	-/-	-/-	-/-	w/w	+/+	4/4
10-1414	-/-	-/-	-/-	-/-	-/-	-/-	-/-	w/w	w/w	4/4
10-1628	-/-	-/-	-/-	-/-	-/-	-/-	-/-	w/w	w/+	4/4
11-1343	-/-	-/-	-/-	-/-	-/-	-/-	-/-	w/w	w/+	3/3
11-1344	-/-	-/-	-/-	-/-	-/-	-/-	-/-	+/+	w/+	3/3
CBS 8517	-/-	-/-	-/-	-/-	-/-	-/-	-/-	w/w	w/+	4/4
CBS 2900	-/-	-/-	-/-	-/-	-/-	-/-	-/-	w/w	w/+	1/1
CBS 3014	-/-	-/-	-/-	-/-	-/-	-/-	-/-	w/w	w/w	2/2

*Detected enzyme activity and H_2S production on the 4th day/on the 7th day.

+, activity detected; -, activity not detected; w, medium activity

Arbitrary scaling: (1) cream, (2) light brown, (3) brown, (4) dark brown, (5) black colour of the yeast colony.
